# Severe hypercalcemia and a pelvic brown tumor in an adolescent with primary hyperparathyroidism: a case report

**DOI:** 10.1186/s12887-020-02445-7

**Published:** 2020-12-05

**Authors:** O. Legault, M. Inman, N. Moolman, S. Wiebe, A. Poulin, M. A. Nour

**Affiliations:** 1 Department of Pediatrics, College of Medicine, Royal University Hospital, University of Saskatchewan, 103 Hospital Drive, SK S7N 0W8 Saskatoon, Canada; 2 Department of Surgery, College of Medicine, University of Saskatchewan, 103 Hospital Drive, S7N 0W8 Saskatoon, SK Canada; 3 Department of Medical Imaging, College of Medicine, University of Saskatchewan, 103 Hospital Drive, S7N 0W8 Saskatoon, SK Canada; 4 Department of Pathology, College of Medicine, University of Saskatchewan, 103 Hospital Drive, S7N 0W8 Saskatoon, SK Canada

**Keywords:** Hyperparathyroidism, Osteitis fibrosa cystica, Hypercalcemia, Hungry bone syndrome, Case report

## Abstract

**Background:**

Primary hyperparathyroidism may present in a myriad of manners, varying from an incidental asymptomatic biochemical finding to gastrointestinal, psychiatric, renal, and bone manifestations. While hyperparathyroidism remains a rare diagnosis in the pediatric population, the initial approach to diagnosis and management of hypercalcemia in children is imperative for the general pediatrician. Herein, we describe an adolescent who presented with a lytic bone lesion and severe, symptomatic hypercalcemia due to primary hyperparathyroidism.

**Case presentation:**

A 14-year-old male presented with vomiting, constipation, abdominal pain, and lethargy. He had an elevated total corrected calcium of 4.3 mmol/L. He was found to have a large pelvic lytic tumor consistent with a brown tumor due to primary hyperparathyroidism. He received pharmacologic therapy for stabilization of his hypercalcemia, including intravenous saline, intravenous bisphosphonates, and calcitonin. He subsequently received definitive therapy via parathyroidectomy and his post-operative course was complicated by hungry bone syndrome. Long-term follow-up has found full resolution of the lytic lesion and restored calcium homeostasis.

**Conclusions:**

We present this case to highlight the possible presentations of hypercalcemia and hyperparathyroidism that are essential for a general pediatrician to recognize to ensure prompt diagnosis and management. Evaluation for hypercalcemia should be considered in patients with suggestive symptoms and physical exam findings. To our knowledge, this patient represents the first reported pediatric case of a pelvic brown tumor in an adolescent. While the multi-systemic complications of hyperparathyroidism may be quite severe, swift and appropriate management may mitigate these clinical outcomes.

## Background

Primary hyperparathyroidism is a rare entity in the pediatric population. The presentation is commonly an incidental biochemical finding with asymptomatic hypercalcemia. If symptomatic, patients may describe fatigue, headache, nausea, abdominal pain, vomiting and polydipsia. End organ complications are also described, including nephrocalcinosis, renal impairment and osteoporosis.[1] Bone manifestations of hyperparathyroidism typically present as a silent preferential loss of bone at cortical sites with reduced bone mineral density and osteoporosis, while *osteitis fibrosa cystica* or ‘brown tumors’ are a rare manifestation.[2] We report here the case of an adolescent male who presented with severe, symptomatic hypercalcemia due to primary hyperparathyroidism and a large lytic lesion of the pelvis consistent with a brown tumor. This represents, to our knowledge, the first reported pelvic brown tumor in a pediatric patient. This case highlights the spectrum of disease severity and complications of hyperparathyroidism for the general pediatrician to be mindful of. The principal aspects of the pharmacological and surgical treatment are also be reviewed.

## Case presentation

### Clinical Presentation

A 14-year-old previously healthy male was admitted with a 3-week history of daily emesis, constipation, and lethargy. He had a decreased appetite and 3 kg weight loss in the preceding weeks. He described vague periumbilical pain and his family noticed a recent depressive change in his mood, including a fatigued appearance, low mood, and withdrawn behaviour. He had no joint or bone pain and denied fever or night sweats. Past medical history was unremarkable. He was on no medications. The family had recently immigrated from the Philippines one-week prior to the onset of symptoms. On exam, he appeared unwell and cachectic with a weight of 37 kg (-2.1 standard deviation (S.D.)) and a body mass index (BMI) of 14.1 kg/m² (<-3 S.D.). He was alert, oriented and cooperative. Neurological exam was unremarkable with normal deep tendon reflexes, bilateral flexor plantar reflexes, a normal gait and normal strength. A large, irregular, tender, mobile abdominal mass of 10 × 5 cm was palpable in the left lower quadrant.

### Diagnostic Assessment

Initial laboratory testing demonstrated severe hypercalcemia and hypophosphatemia, with a corrected total serum calcium of 4.30 mmol/L (N: 2.14–2.65 mmol/L) and a phosphate of 0.58 mmol/L (N: 0.74–1.52 mmol/L). Initial serum intact parathyroid hormone (PTH) was elevated at 51.70 pmol/L (N: 1.30–6.80 pmol/L), later peaking at 70.2 pmol/L. Additional biochemical evaluation on presentation is provided in Table 1.

**Table 1:** Summary of biochemical investigations at presentation

Test	Result	Reference Interval
Total Calcium (mmol/L)	4.13	2.14–2.65
Corrected Calcium (mmol/L)	4.30	2.14–2.65
Phosphate (mmol/L)	0.58	0.74–1.52
Magnesium (mmol/L)	0.75	0.66–1.07
Albumin (g/L)	34	35–52
Alkaline Phosphatase (U/L)	328	40–150
PTH (pmol/L)	51.7	1.3–6.8
25-OH Vitamin D (nmol/L)	45.4	70–250
1,25(OH)_2_ Vitamin D (pmol/L)	197	64–226

*PTH* Parathyroid hormone level

**Fig. 1:**
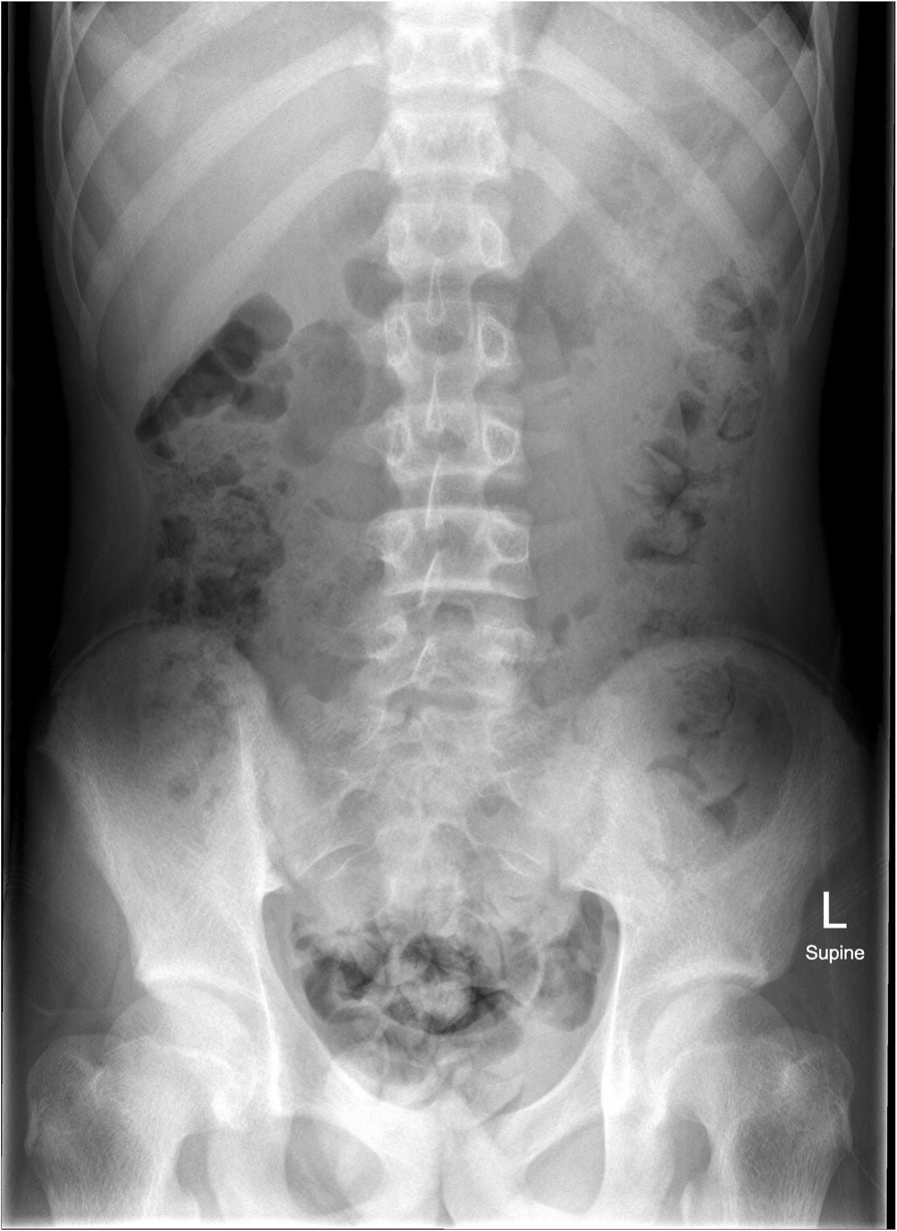
Radiograph at presentation demonstrating the well-defined low-density lesion (Brown Tumor) within the left iliac bone

**Fig. 2:**
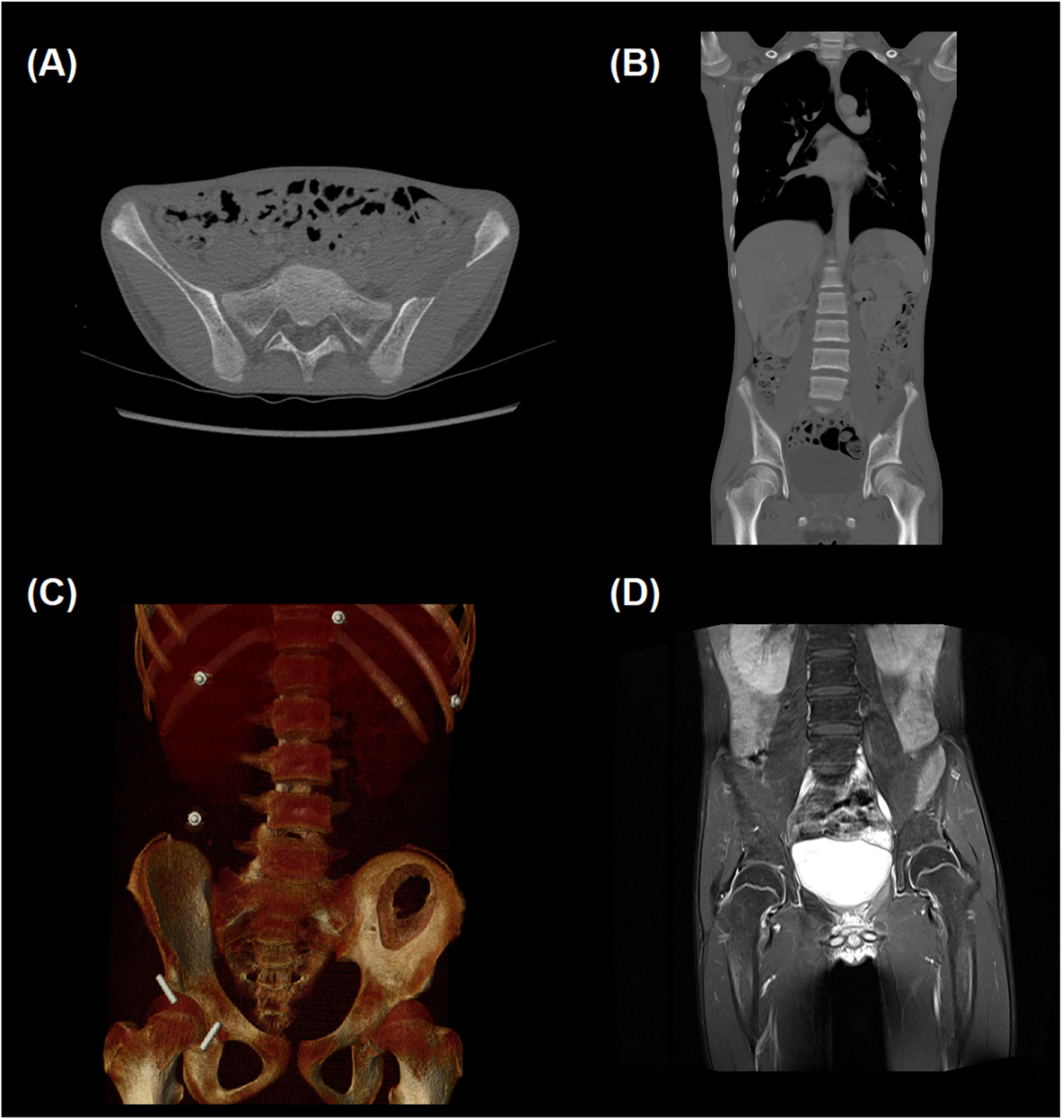
**a** Axial, **b** Coronal (bone window), and **c** 3-dimensional reconstruction CT imaging demonstrating a lytic lesion in the left iliac bone. **d** Coronal T2 MR image demonstrating the increased T2 signal in the tissue within the lytic bone lesion

**Fig. 3:**
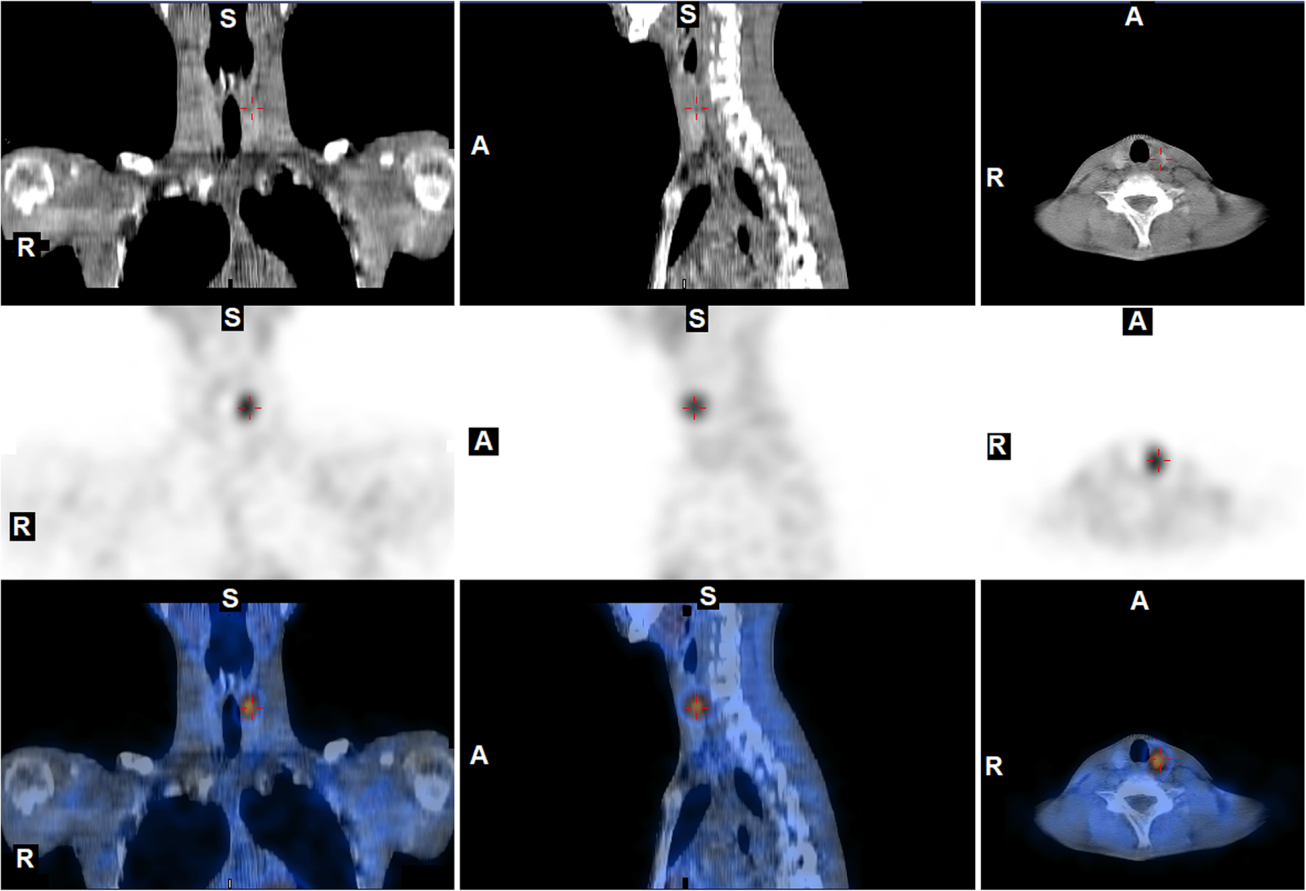
Nuclear Medicine technetium 99 m Sestamibi parathyroid scan with supplemental SPECT-CT imaging demonstrating a solitary focus of increased radiotracer uptake. From top to bottom, the rows of images demonstrate CT images, images reconstruction with attenuation correction (IRAC), and hybrid CT-IRAC SPECT-CT images, respectively. From left to right, the columns of images depict coronal, sagittal, and transaxial images, respectively. (A: anterior; R: right; S: superior)

Given the palpable mass and reported symptoms of emesis and constipation, a plain film abdominal radiograph was performed on admission to evaluate the abdominal mass and assess for possible obstruction. This identified large fecal loading of the colon and a lytic lesion within the left iliac wing (Fig. 1). Subsequent computed tomography (CT) and magnetic resonance imaging (MRI) imaging showed the lesion arising within the bone, measuring 13 × 5.1 × 5.8 cm and a scalloped appearance with a T2 intense soft tissue component slightly projecting beyond the anterior table of the iliac bone (Fig. 2). Thyroid ultrasound and Nuclear Medicine technetium 99 m Sestamibi scan were consistent with the identification of a solitary left superior parathyroid adenoma (Fig. 3).

No further bony lesions were identified with full body skeletal survey radiographs and scintigraphy. Renal ultrasound was unremarkable with no nephrocalcinosis nor nephrolithiasis. Bone mineral densitometry (BMD) demonstrated lumbar spine, whole body and left distal forearm Z-scores of -0.2, -1.1, and − 1.7, respectively.

**Fig. 4:**
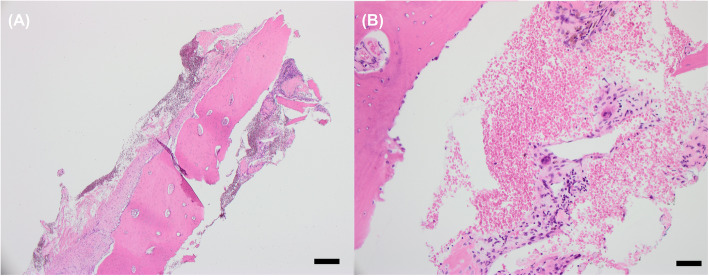
Core needle biopsy of left iliac crest. **a** Viable bone with reparative changes at H&E 4X magnification. (Black bar denotes 500 μm at this resolution) **b** Rare osteoclast-like giant cells, hemorrhage, and hemosiderin are present in a background of spindle cells consistent with brown tumor at H&E 20X magnification. (Black bar denotes 50 μm at this resolution)

CT guided core needle biopsy of the iliac bone lesion was performed and underwent histological assessment demonstrating viable bone surrounded by moderately cellular spindle cells with hemosiderin, hemorrhage, and rare osteoclast-like giant cells, consistent with osteitis fibrosa cystica (Fig. 4).

### Therapeutic Intervention

**Fig. 5:**
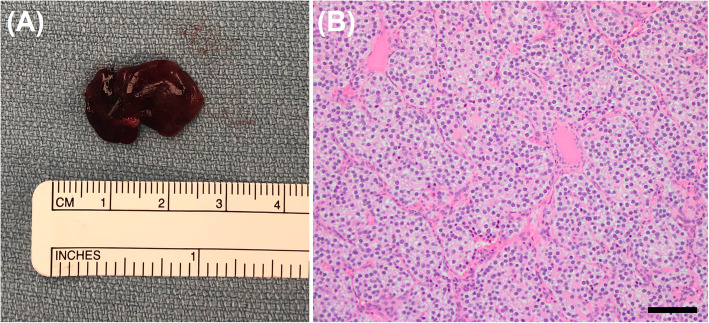
**a** Excised left superior parathyroid gland adenoma measuring 2.0 × 1.6 × 0.4 cm. **b** Histological specimen demonstrating nests of proliferating parathyroid cells lacking intermixed adipose tissue, consistent with a parathyroid adenoma (H&E 20X magnification, black bar denotes 100 μm)

Prior to surgical treatment, hypercalcemia was treated with intravenous (IV) normal saline, multiple doses of subcutaneous (SC) calcitonin and a single dose of IV Zoledronic acid (0.025 mg/kg). The patient underwent surgical excision of a left superior parathyroid gland (2.0 × 1.6 × 0.4 cm; 0.9 g) with histology consistent with a parathyroid adenoma (Fig. 5*)*. The postoperative course was complicated by prolonged, recurrent hypocalcaemia with a total serum calcium nadir of 1.98 mmol/L, consistent with “hungry bone syndrome”. He required IV and oral replacement of calcium for 6 days. He was later discharged to home with oral supplementation of calcium carbonate and cholecalciferol.

### Follow-up and outcomes

In the subsequent 8 months post-parathyroidectomy, his weight increased 17 kg to 54 kg (-0.4 S.D.) and BMI to 19.8 kg/m² (0.0 S.D.). Serum biochemistry was maintained with gradual weaning and discontinuation of supplementation of calcium and vitamin D; serum total calcium 2.43 mmol/L, phosphate 1.66 mmol/L, and PTH 6.2 pmol/L. Repeat pelvic MRI within 12-months post-parathyroidectomy demonstrated complete resolution of pelvic lesion.

## Discussion and conclusions

Hypercalcemia is a relatively rare presentation in the pediatric population, however, an approach to the differential diagnosis and awareness of initial management steps is important for the general pediatrician. While hypercalcemia in neonates and infants includes a number of genetic and iatrogenic possibilities, the diagnosis in the adolescent – much like in adults – is most commonly due to one of three aetiologies: hyperparathyroidism, malignancy or immobilization.[3] Given the large palpable abdominal mass found in our patient, initial assessments were highly suspicious for an underlying malignancy. However, biochemical and radiologic evaluation confirmed the diagnosis of hyperparathyroidism and proved the abdominal mass to be due to constipation and fecal impaction secondary to severe hypercalcemia.

Initial management for all patients with severe symptomatic hypercalcemia must begin with IV fluid administration for dilution and subsequent renal excretion of excess calcium. Hypercalcemia can induce dehydration through impaired oral intake and is compounded by possible development of nephrogenic diabetes insipidus. Additional therapies to reduce serum calcium levels may include antiresorptive agents (i.e. bisphosphonates), calcitonin, forced calciuresis, and corticosteroids. Bisphosphonates inhibit osteoclastic activity and hinder hydroxyapatite breakdown, a mineral composed of calcium and phosphate. Calcitonin inhibits bone resorption and forces renal excretion of calcium, but its effect is limited due to development of tachyphylaxis within days of initial exposure. Facilitating calciuresis through diuretic use, such as furosemide, should be delayed until intravascular volume has been replaced. The use of corticosteroids is potentially beneficial in select etiologies of hypercalcemia that are primarily mediated by excessive production of activated vitamin D (ex. granulomatous diseases).

This patient highlights several unique aspects to the presentation of hyperparathyroidism. Primary hyperparathyroidism is characterized by the excessive secretion of PTH by one or multiple parathyroid glands. PTH promotes bone catabolism of cortical bone and bone anabolism of cancellous bone.[4] This was demonstrated here with a relative reduction in BMD of the distal 1/3 forearm – a primarily cortical site – and minimal impact on BMD of the lumbar spine – predominantly trabecular. Osteolytic lesions such as brown tumors manifest in severe and prolonged courses of hyperparathyroidism and excessive osteoclastic activity. Renal involvement from hyperparathyroidism is more frequent than bone involvement and manifests with hypercalciuria, nephrocalcinosis, and nephrolithiasis. Neuropsychological symptoms are also associated with hypercalcemia and include nonspecific complaints such as weakness, fatigue, depression and anxiety as well as complaints of decreased memory and concentration, loss of initiative, irritability, and disturbed sleep.[5].

The diagnosis of a brown tumor is primarily clinical through the presence of either a single or multiple well-demarcated osteolytic lesions in the setting of hyperparathyroidism and hypercalcemia. Histologic features of the tumor are non-specific and may resemble other giant cell tumors [6, 7] and is the result of an increased activity of osteoclasts replacing bone with reactive fibrous tissue.[8] Brown tumors received their name due to the brown tissue appearance resulting from microhemorrhages of interstitium, causing hemosiderin deposits. The incidence of brown tumors are quite rare, occurring in approximately 2% of adult patients suffering from any form of hyperparathyroidism and commonly affect the mandible, clavicle, ribs, and pelvis.[9–11]. While most presentations of hyperparathyroidism are asymptomatic in occidental countries, skeletal manifestations are seen more commonly in countries such as India, Iran, Saudi Arabia and Thailand.[12] Reports of brown tumors are exceedingly rare in the pediatric patient and the few cases described were reported in children 14 to 18 years of age with osteolytic lesions presenting in the skull, mandibula, scapula, clavicles, ribs, sternum, vertebrae, and long bones. [13–17] To our knowledge a pelvic manifestation of brown tumor has never been reported in a pediatric patient, however cases have been reported in adults 39 to 69 years of age. [11, 18]

Definitive therapy for primary hyperparathyroidism and brown tumors is surgical through parathyroidectomy. In cases of severe, prolonged hyperparathyroidism, there is a significant postoperative risk of developing hungry-bone syndrome.[19] This results in acute hypocalcemia from rapid deposition of calcium into bone. As this hypocalcemia is driven by autonomous ‘hungry’ osteocytes directing osteoblastic deposition of calcium, the mainstay of therapy for hungry-bone syndrome is replacement of calcium via IV and oral routes. Bisphosphonate therapy was also utilized in order to potentially reduce the expected risk of post-operative hungry bone syndrome.[20] Given bisphosphonates primarily inhibit osteoclastic bone resorption, the mechanism of prevention of hungry bone syndrome is poorly understood, however is thought to be the result of a transient inhibition of bone mineralization. [21] Therapy may further be augmented through the use of active metabolites of Vitamin-D (Calcitriol) to maximize enteric absorption of calcium. [19, 22]

## Conclusions

With a wide spectrum of presenting features, severe hyperparathyroidism may result in significant pathology, multisystemic involvement with end-organ damage requiring urgent recognition and therapy. Its mimicry of malignancy requires a thorough clinical review and for patients presenting with severe disease, a combination of acute pharmacologic treatment to reduce hypercalcemia followed by definitive surgical intervention are required for optimization of short- and long-term outcomes. This case reports an extremely rare manifestation of chronic hyperparathyroidism, pelvic brown tumor, and exemplifies the importance of clinical recognition of hypercalcemic symptoms to facilitate prompt diagnosis and acute management.

## Data Availability

Not applicable.

## Abbreviations

BMI: Body mass index
CT: Computed tomography
IV: Intravenous
MRI: Magnetic resonance imaging
PTH: Parathyroid hormone
S.D.: Standard deviation
